# Coexistence of TEMPI syndrome and leukocytoclastic vasculitis successfully treated with autologous stem cell transplantation

**DOI:** 10.1002/jha2.561

**Published:** 2022-10-10

**Authors:** Davide Nappi, Martina Tauber, David Sykes

**Affiliations:** ^1^ Hematology and Bone Marrow Transplantation Bolzano Hospital Bolzano Italy; ^2^ Department of Pathology Bolzano Hospital Bolzano Italy; ^3^ Massachusetts General Hospital Center for Regenerative Medicine Boston Massachusetts USA

**Keywords:** angiogenesis, erythrocytosis, inflammation, MGUS, multiple myeloma

## Abstract

The TEMPI syndrome is a very rare paraneoplastic syndrome associated with plasma cell dyscrasia and monoclonal gammopathy. First described in 2011, the pathophysiology of TEMPI syndrome is still unknown. Essential for diagnosis is to recognize the five clinical findings: telangiectasias, erythrocytosis and elevated serum erythropoietin, monoclonal gammopathy, perinephric fluid collection, and intrapulmonary shunting. Here we report a case of a woman with the coexistence of TEMPI and leukocytoclastic vasculitis, shedding light on a possible common inflammatory pathway involved in the pathogenesis of the syndrome.

1

TEMPI syndrome belongs to the recently defined category of monoclonal gammopathy with clinical significance (MGCS), a heterogeneous group of clonal plasma‐cell diseases whose signs and symptoms are strictly related to the presence of an M‐protein [1]. First described in 2011, the TEMPI acronym denotes (1) telangiectasias, (2) elevated erythropoietin and erythrocytosis, (3) monoclonal gammopathy, (4) perinephric fluid collections, and (5) intrapulmonary shunting, that are the hallmark of this very rare paraneoplastic syndrome [2, 3].

In 2006, a 40‐year‐old woman was seen in the clinic for a diagnosis of idiopathic erythrocytosis. JAK2 V617F and JAK2 exon 12 testings were negative, bone marrow biopsy showed no evidence of a myeloproliferative disorder, and other possible causes of secondary erythrocytosis were excluded. Given that her hematocrit value has risen to >55%, she had been subjected regularly to therapeutic phlebotomy.

A monoclonal gammopathy of undetermined significance (MGUS) IgA‐Lambda of 3 g/dl was incidentally detected in 2010. She was not felt to meet the criteria for multiple myeloma (MM) given the absence of CRAB criteria and the finding of <10% of plasma cells in the bone marrow, but with an unusual hypercellular background (Figure 1A). However, FISH analysis of her plasma cells unexpectedly revealed two high‐risk features: del17p and t(4;14). POEMS syndrome was excluded by the absence of polyneuropathy, the absence of osteosclerotic lesions, and a normal level of serum vascular endothelial growth factor (VEGF)‐A.

**FIGURE 1:**
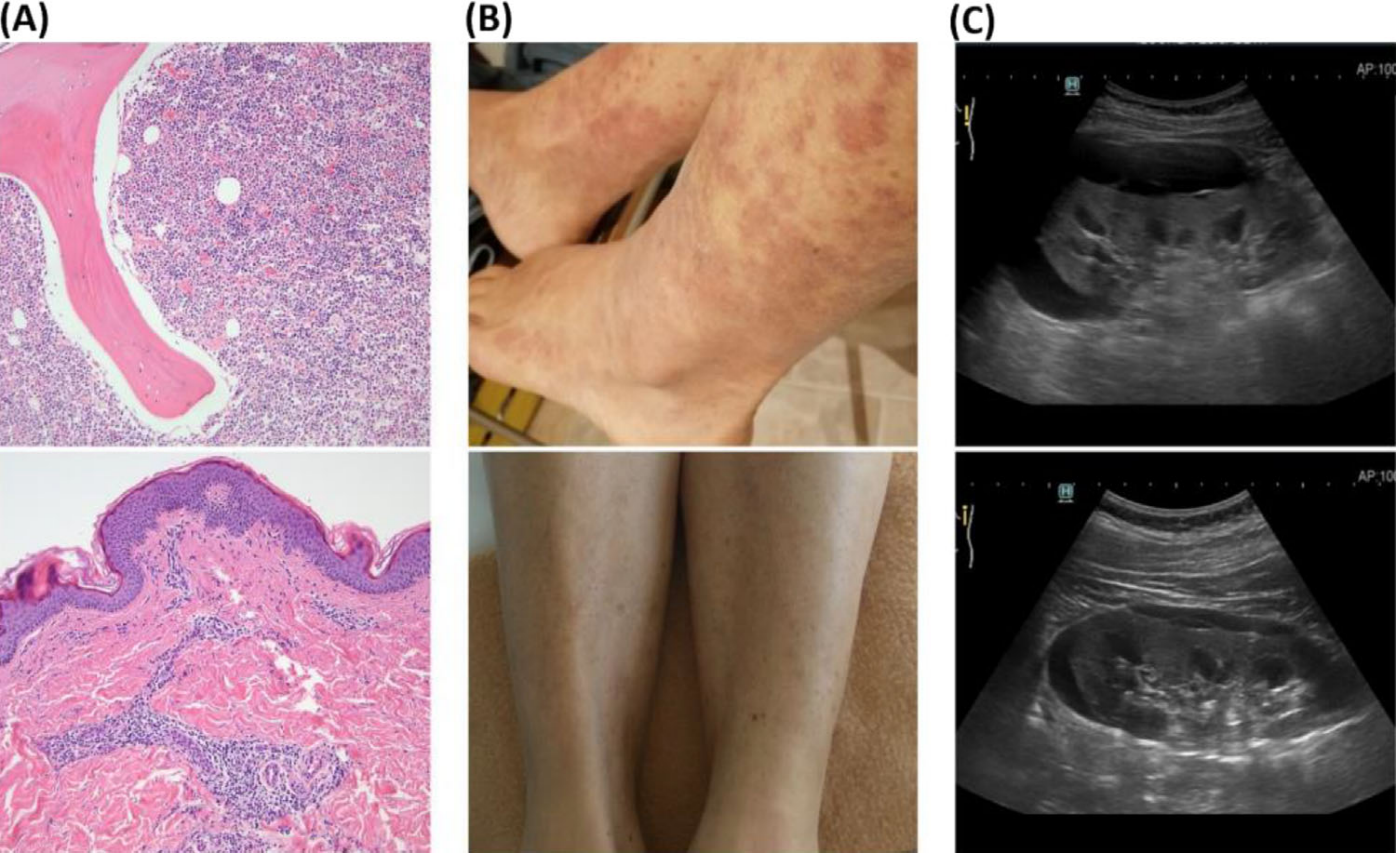
(A) Histopathological findings: bone marrow biopsy (above) showing increased cellularity with myeloid expansion and skin biopsy (below) of vasculitic lesions with evident perivascular lymphocytic infiltration. (B) Lower limbs leukocytoclastic vasculitis before and after treatment. (C) Ultrasound image of perinephric fluid collection on the left kidney before and after treatment

The patient began to develop an atypical dermatologic syndrome comprised of telangiectasias, on the skin of her face and trunk, as well as a leukocytoclastic vasculitis that was most prominent on her lower limbs (Figure 1B). As part of her workup, screening for anti‐neutrophil cytoplasmic antibodies and anti‐nuclear antibody was negative. A skin biopsy was performed (Figure 1A) which revealed an inflammatory small vessel perivascular infiltrate, light C3 perivascular dermal deposition, and an absence of IgA deposition.

In 2021, the patient reported a worsening in her clinical condition as evidenced by dyspnea that was accompanied by hypoxia and resting oxygen saturation of 90%–95%. Her telangiectasias were more numerous and more prominent and she had developed hypertension. Given the suspicion of TEMPI syndrome, an abdominal ultrasound was performed, revealing bilateral perinephric fluid collections (maximum thickness 2.5 cm on the right), with a mild compression pattern on the underlying parenchyma. (Figure 1C). An echocardiogram with agitated saline contrast was suggestive of microscopic intrapulmonary shunting. Her serum erythropoietin level was dramatically elevated at 2800 IU/ml (Figure 2). She was given the diagnosis of TEMPI syndrome with concomitant leukocytoclastic vasculitis and adverse plasma cell cytogenetic features.

**FIGURE 2:**
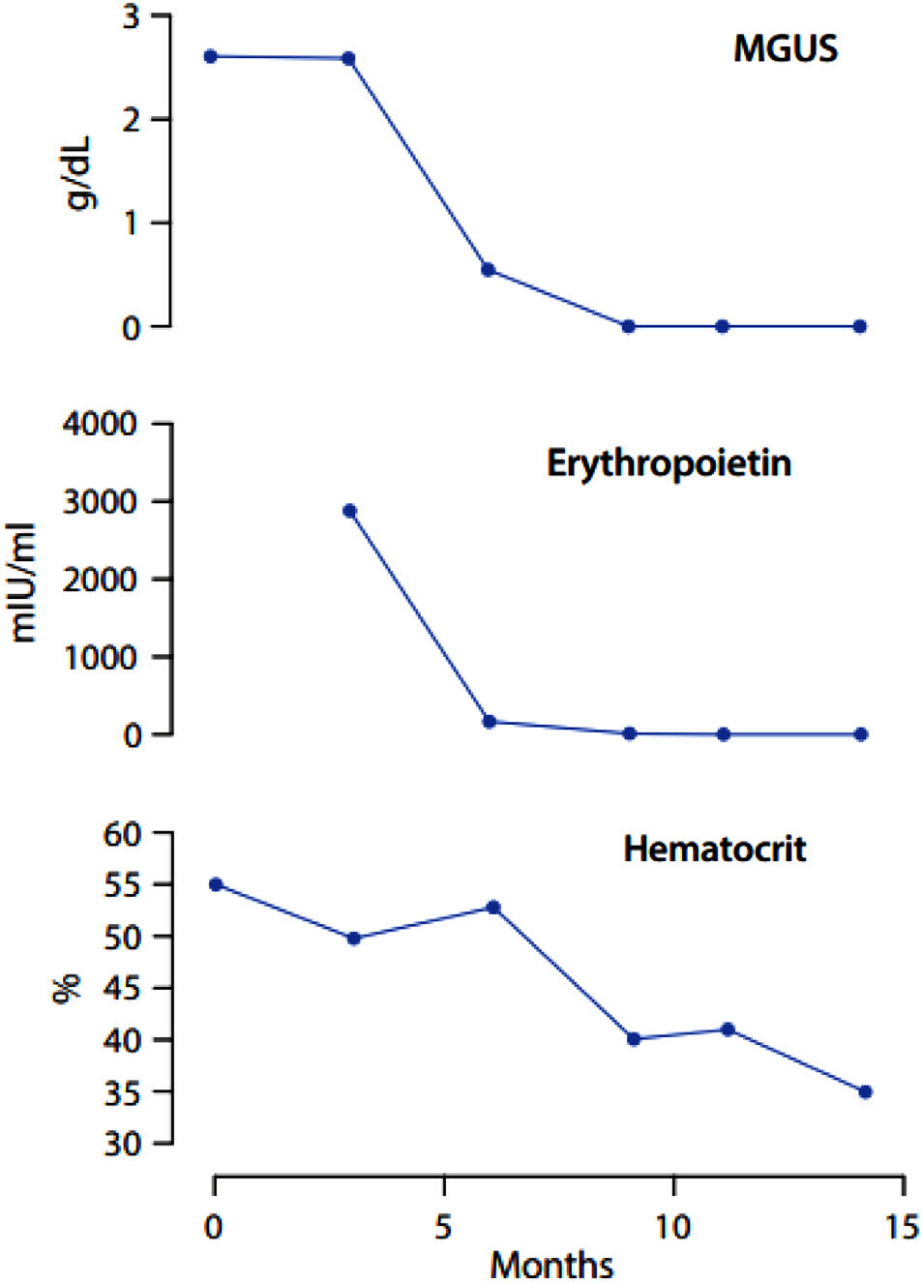
Disease parameters (monoclonal gammopathy of undetermined significance (MGUS), serum erythropoietin, and hematocrit) measured at baseline and over treatment. MGUS and erythropoietin levels disappeared and normalized, respectively, after 1 cycle of treatment; hematocrit had a slower response

Given her young age, progressive symptoms, disabling hypoxia, and adverse cytogenetic features, we opted for medical treatment directed at her clonal plasma cell dyscrasia. She received five cycles of bortezomib‐cyclophosphamide‐dexamethasone therapy, obtaining a complete hematological, laboratory, and clinical response. The rapid drop in her monoclonal component, hematocrit, and serum erythropoietin was followed by the total resolution of both her telangiectasias and leukocytoclastic vasculitis, confirming a dramatic response to plasma cell‐directed therapy (Figure 2). Following the success of the initial treatment, and with the goal of obtaining a long‐lasting remission, she underwent consolidation treatment with an autologous stem cell transplantation (ASCT) in February 2022. The ASCT was tolerated remarkably well and the patient is currently in complete remission, with the total disappearance of skin lesions and remarkable thinning of perinephric fluid (Figures 1 and 2).

As of May 2022, approximately 30 cases of TEMPI syndrome have been reported worldwide [4], and continued information about the various clinical features and patient outcomes to treatments is emerging. Patients typically show an earlier average age of onset of symptoms than in MM, with an underlying bone marrow plasma cell burden ≤10% with predominant MGUS or smoldering MM features (only one case has reported overt MM). Patients with TEMPI syndrome generally have a dramatic response to plasma cell‐directed treatment which has included bortezomib, lenalidomide, and daratumumab [5, 6, 7, 8, 9]. As with our case, some authors have reported that treatment with ASCT has yielded long‐term responses [10].

Despite the effectiveness of treatment that eradicates the plasma cell clone from the bone marrow, strongly suggesting an etiological pathophysiologic link, it is still unknown how the plasma cell and/or the monoclonal gammopathy can induce the signs and symptoms of TEMPI syndrome. In MGCS, the pathogenetic role of the M‐protein, secreted by clonal plasma cells, varies from direct autoantibody activity to cytokine‐mediated effects, though in the TEMPI syndrome, the actual causal link remains a matter of debate.

A stimulus of abnormal angiogenesis could be the pathogenetic linker between cutaneous telangiectasias and “microscopic” intrapulmonary shunt [3]. TEMPI syndrome shares some of the neoangiogenesis‐related symptoms with POEMS (erythrocytosis, hemangiomas), that are VEGF‐mediated in POEMS but not in TEMPI [11]. Whole‐genomic sequencing analysis of a small sample of TEMPI patients suggested a potential central role of the pleiotropic cytokine Macrophage migration Inhibitory Factor (MIF) as the direct and indirect molecular mediators in triggering the TEMPI features by promoting a proangiogenic stimulus via HIF‐1a and erythropoietin secretion [12]. MIF is also a pivotal pleiotropic cytokine in the pathogenesis of rheumatic and inflammatory diseases, as well as in vasculitic diseases [13, 14, 15].

Ours is the first case report where TEMPI syndrome is associated with leukocytoclastic vasculitis. The etiology of the two conditions appears to be linked, as suggested by the temporal worsening of both clinical conditions as well as dramatic responses to treatment. This case sheds light on a possible pathogenetic role of inflammatory cytokines in the genesis of TEMPI syndrome. A previous report of a systemic autoimmune disease (rheumatoid arthritis) with pronounced microvascular features highlighted a potential link between TEMPI and abnormal inflammatory response. We hypothesize that the same inflammatory and neoangiogenetic pathways may underly both TEMPI and the vasculitis in this patient, with an unclear role of her monoclonal gammopathy.

## CONFLICT OF INTEREST

The authors declare they have no conflicts of interest.

## FUNDING INFORMATION

The authors received no specific funding for this work.
